# Constraints influencing sports wheelchair propulsion performance and injury risk

**DOI:** 10.1186/2052-1847-5-3

**Published:** 2013-03-28

**Authors:** Emily Churton, Justin WL Keogh

**Affiliations:** 1 Sport Performance Research Institute New Zealand, School of Sport and Recreation, AUT University, Private Bag 92006, Auckland 1142, New Zealand; 2 Bond University Research Centre for Health, Exercise and Sports Sciences, Faculty of Health Sciences and Medicine, Bond University, Gold Coast 4229, Australia; 3 Faculty of Science, Health, Education and Engineering, University of the Sunshine Coast, Sippy Downs, QLD, Australia

**Keywords:** Adapted physical activity, Biomechanics, Constraints-led approach, Dynamical systems theory, Paralympic sport

## Abstract

The Paralympic Games are the pinnacle of sport for many athletes with a disability. A potential issue for many wheelchair athletes is how to train hard to maximise performance while also reducing the risk of injuries, particularly to the shoulder due to the accumulation of stress placed on this joint during activities of daily living, training and competition. The overall purpose of this narrative review was to use the constraints-led approach of dynamical systems theory to examine how various constraints acting upon the wheelchair-user interface may alter hand rim wheelchair performance during sporting activities, and to a lesser extent, their injury risk. As we found no studies involving Paralympic athletes that have directly utilised the dynamical systems approach to interpret their data, we have used this approach to select some potential constraints and discussed how they may alter wheelchair performance and/or injury risk. Organism constraints examined included player classifications, wheelchair setup, training and intrinsic injury risk factors. Task constraints examined the influence of velocity and types of locomotion (court sports vs racing) in wheelchair propulsion, while environmental constraints focused on forces that tend to oppose motion such as friction and surface inclination. Finally, the ecological validity of the research studies assessing wheelchair propulsion was critiqued prior to recommendations for practice and future research being given.

## Introduction

Wheelchair sports such as basketball, rugby, tennis and racing e.g. 100 m through to the Marathon, are becoming increasingly popular for many athletes with disabilities and spectators, with the pinnacle for these athletes being the Paralympic Games. A potential issue for many wheelchair athletes is how to maximise the training-related gains in performance while minimising the risk of injuries [1-3]. Upper limb injuries, particularly to the shoulder appear common in wheelchair athletes due to the accumulation of stress placed on this joint during activities of daily living, training and competition [4-8].

This narrative review/current concept article aims to: 1) use the constraints-led approach of dynamical systems theory to identify key constraints affecting wheelchair propulsion; and 2) critically evaluate a sub-set of relevant articles from the research literature on hand rim wheelchair propulsion during sporting activities; with the overall goal being to propose ways in which to improve wheelchair sporting performance and reduce injury risk. For the purposes of this review, performance measures can be viewed as direct (time to complete a set distance in a race, distance thrown during throwing events etc.) or indirect (VO_2_peak, strength, muscular power etc.). While injury rates are often defined as the number of injuries per 1000 hours of activity, no studies have compared the injury rates of Paralympic athletes under different forms of constraint. Thus, we used injury risk which is much harder to define. In this paper, insight into injury risk was mainly provided by the nature of the internal and external forces acting upon the body. As we found no studies that have directly utilised the dynamical systems approach to interpret their data in Paralympic athlete studies, we therefore thought it appropriate to conduct a narrative review of the literature for how common alterations to the three levels of constraints under which wheelchair locomotion is performed may alter performance and/or injury risk. While numerous organism, task and environmental constraints could be examined in such a narrative review of the literature, we have focused on selected examples of each of these constraints that we feel have the most relevance to these athletes. Specifically, organism constraints examined include player classifications, wheelchair setup, training and intrinsic injury risk factors. Task constraints examined the influence of locomotion (court sports vs racing) at various speeds, while environmental constraints focused on forces that tend to oppose motion such as friction and surface inclination. Finally, the ecological validity of the research studies assessing wheelchair propulsion was critiqued prior to recommendations for practice and future research being given.

## Research methods

Relevant literature was found by inputting the keywords *dynamic systems, constraints-led approach, upper limb biomechanics, wheelchair sport*, and *hand rim propulsion* in the following databases EBSCO Host, SportDiscus, Medline, Science Direct and Google Scholar. Reference lists of all identified references were examined in order to find more relevant references. To be included in the review, articles needed to be in peer-reviewed journals and involve participants who used wheelchairs in their daily living and sporting activities. As a narrative review similar to that of Keogh [9], this manuscript has not systematically reviewed all of the literature, but cited and examined the most relevant literature pertaining to the three levels of constraint so to more critically examine ways to improve performance and reduce injury risk in wheelchair sports.

### Dynamical systems theory

**Figure 1:**
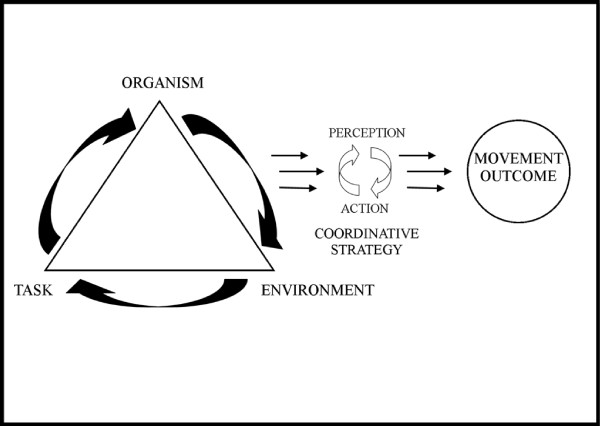
An example of how the three levels of constraints interact and create functional variability (adapted from Davids et al., 2008).

The constraints led approach views the outcome and coordination of movement as the result of the manner in which the three levels of constraints (environmental, task and organism) under which the movement is performed interact [10]. This is depicted pictorially in Figure 1, showing how the interaction of the three levels constraints may impact on performance and injury risk. Studies using the constraints led approach to investigate human movement have focused on able-bodied sport or injury [11-14]. Currently, no such studies appear to have been conducted on wheelchair propulsion, although this approach has been used in one gait study of individuals with a lower limb amputation [15].

Organism constraints refer to unique characteristics a person has, such as level of disability, that influence the manner in which their movements are performed. In Paralympic wheelchair sports, the wheelchair-user interface can be viewed as the organism through which performance and injury risk need to be evaluated. Environmental constraints are global extrinsic factors that can impact movement co-ordination whereas task constraints are more specific to performance and include task goals, specific rules associated with a sport and activity related implements or tools [10]. The variety of disabilities and wheelchair designs (and hence organism-level constraints) seen within- and between-Paralympic events and athletes suggests that subtle differences in the optimal coordinative strategy and injury risk would exist for each athlete, even if the environmental and task constraints they encounter are identical.

The concept of functional movement variability is another dynamical systems theory principle that may be relevant to coaches, sports science and medicine staff who work with Paralympic athletes. Functional variability can be viewed as the ability to adopt a flexible (variant) movement control strategy that enables the athlete to perform at a consistently high level even when the interaction of the three levels of constraints have changed [13]. Based on this view, the minimisation of all movement variability (i.e. absolute invariance of technique) may be sub-optimal and lead to increased injury risk as the same anatomical structures would have to produce and/or tolerate the muscular and external forces, respectively. This view is supported by several studies involving able-bodied athletes, whereby greater performance or reduced injury risk was associated with higher levels of functional variability [12-14].

### Organism constraints

Constraints discussed in this section reflect those factors influencing the wheelchair-user interface. Selected articles on player classification, wheelchair set-up, training and intrinsic injury factors and their impact on wheelchair locomotion are examined.

#### Player classification

The wheelchair sports of basketball, rugby, tennis and racing are all included in the Paralympics and each sport has functional classifications which takes into account each person’s organism constraints, i.e. the extent of their impairment. This is one of the most critical issues in wheelchair sports as the functional classification allows the grouping of players with a similar level of functional capacity based on their ability to perform movements. This aims to eliminate competitive inequalities due to the greater or lesser severity of the impairment of different athletes [16], and gives those with a spinal cord injury or other disabilities, such as polio, cerebral palsy, or amputation, an opportunity to play the sport [17]. The following section will use wheelchair basketball to provide a brief example of the functional classification approach, and how these differences may influence the optimal chair design (to be discussed in the next sub-section), performance role and capabilities as well as injury risk of these athletes. Somewhat similar classification principles are used in the other sports involving wheelchair athletes. For further information on these other sports, the interested reader should consult the International Paralympic Council’s website.

**Figure 2:**
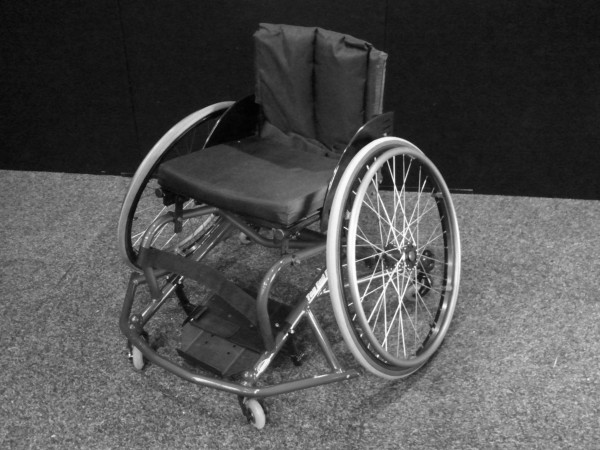
An example of a low point wheelchair basketball wheelchair.

**Figure 3:**
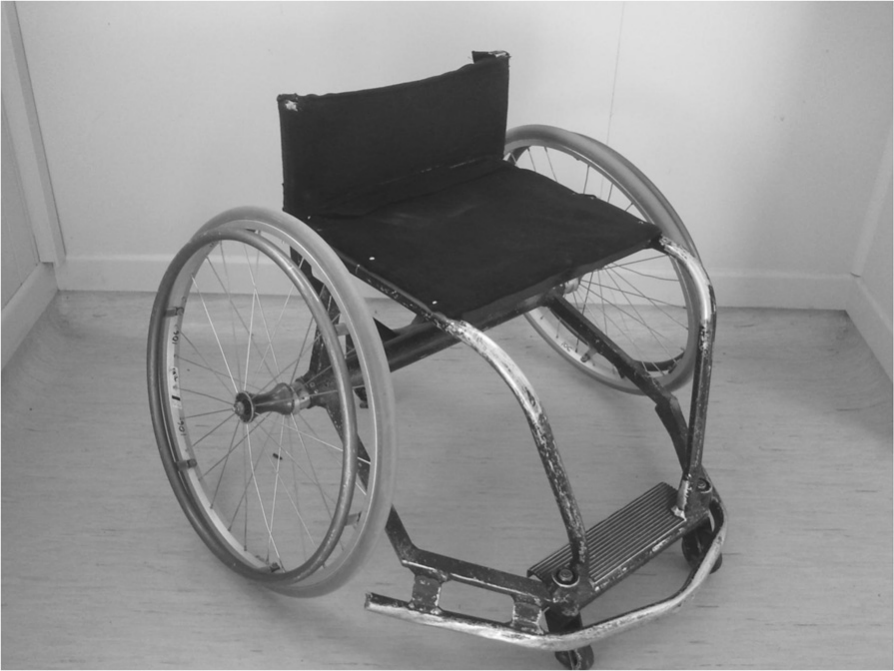
An example of a high point wheelchair basketball wheelchair.

Under the system used by the International Wheelchair Basketball Federation (IWBF), players are categorised based on their physical capacity in terms of playing skills, and the trunk movement and stability observed during pushing, pivoting, shooting, rebounding, dribbling, passing and catching [18]. Wheelchair basketball players are assigned a classification from 1.0 through to 4.5. As low point players (1.0–2.0) have the least physical function and cannot actively stabilise their pelvis, they rely on the external support of the wheelchair for stability and will usually use a seat that is significantly angled downward from front to back (Figure 2). The high point players (3.0–4.5) have more physical function and require little support from the wheelchair as they can actively stabilise their pelvis, meaning they will use a chair with a relatively flat seat (Figure 3) [18]. Further exploration of the wheelchair setup will be provided in the next sub-section.

Very little research has been conducted to examine performance differences in wheelchair team sports athletes of different classifications. Malone and colleagues [19] sought to determine the primary factors associated with successful free throw shooting and if these factors differed between classification groups in wheelchair basketball. All of the free throws taken at one end of the court were recorded at the 1994 Men’s Gold Cup World Wheelchair Basketball Championship, with the analysis conducted on all successful clean shots. Malone et al. [19] categorised all players into one of four groups, so that there were seven clean shots for players in Group I, 16 in Group II, 18 in Group 3 and 26 in Group 4. While many significant differences were observed in the height, angle and speed of release as well as joint kinematics between the groups, tournament statistics indicated that the free throw shooting percentages were very similar across all groups, with the highest percentages being for Group 4 (54%) and the lowest for Group 3 (49%). Based on these results, Malone et al. [19] suggested that the higher classification players did not utilise their theoretical advantages in free throw shooting so that they underperformed in comparison to their lower point colleagues.

Players in different classification categories may also have somewhat different injury risks. According to a study by Reid, Elliot and Alderson [20] involving wheelchair tennis players, those with less trunk control used the upper limb and shoulder muscles to provide a greater percentage of the propulsive power in serving and may also be exposed to higher shoulder joint posterior forces to help resist superior translation of the humeral head during the follow through. If such differences are seen in other Paralympic wheelchair sports, particularly in those sports in which the low point players have similar wheelchair propulsion and throwing/hitting involvement, injury prevention programmes for those with high or low levels of function may need to be more specifically developed so to offset the low pointers greater injury risk.

#### Chair set up

With the increased popularity of wheelchair sports, new wheelchair designs are being continually developed to improve aspects such as acceleration, maximal speed and turning capacity and to prevent tipping over so to fit specific sport needs [21,22]. Wheelchairs can now be altered in many ways and a variety of studies [23-25] have shown that the chair set up can alter propulsion biomechanics in ways that may be beneficial for both sport performance and reducing injury risk.

For example, a comparative study by Coutts [24] involving wheelchair basketball players and wheelchair racers found that the basketball wheelchairs had larger diameter hand rims. Coutts [24] suggested that the greater hand rim diameters of the wheelchair basketball players allowed them greater time to apply propulsive forces (impulses) and hence develop greater levels of acceleration as required due to the stop-start and change of direction nature of wheelchair basketball. This view of Coutts [24] was supported more recently by Costa et al. [23] who examined the relationship between stroke frequency, push time and wheelchair velocity using different hand rim diameters in a class T-52 wheelchair track athlete. They found that when greater torque is needed for rapid speed increases, a larger diameter hand rim could be more effective due to its longer lever arm. However, it must be acknowledged that a larger hand rim may also have some negative consequences. Specifically, larger hand rims may require athletes to apply propulsive torques over larger range of motions. As each athlete may have different upper limb ranges of motion and as extreme ranges of motion during wheelchair propulsion may increase injury risk [4,6], there would likely be a safe upper limit hand rim diameter for different wheelchair athletes and sports.

Lowering the seat, and therefore decreasing the vertical distance between the axle and the shoulder, makes more of the hand rim available for the push cycle, increasing the push angle and contact time between hand and rim. Kotajarvi et al. [25] found that average peak radial and axial force components were also significantly higher in lower seat positions. This may be advantageous as it implies that more force is being directed perpendicular to and toward the axle, respectively in the lowest seat height positions that were tested. However, it is not currently well understood how low the seat should be for different sports and athletes with varying levels of disability and how this might be affected by the diameter of the wheels and hence height of the axle.

**Figure 4:**
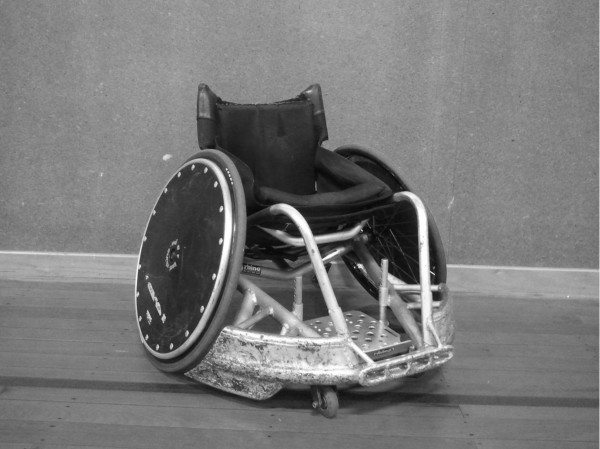
An example of a low point wheelchair rugby wheelchair.

A low seat angle achieved by increasing the wheel camber, as shown by the wheelchair in Figure 4, may also provide advantages for low point players in wheelchair basketball and rugby. Increasing the camber of the wheels will broaden the wheelchair’s base of support, making it harder for opponents to get passed them and also improve stability in contact situations. However, increasing wheel camber does have some disadvantages. Specifically, Faupin et al. [26] found that increased rear-wheel camber resulted in significant increases in residual torque and total muscular power required during the push phase for a given wheelchair velocity and a significant decrease in the mean wheelchair velocity per arm cycle.

As the results of these and other studies provide some indication of the effect of altering certain aspects of chair design, coaches and sport scientists should seek to use such information when determining the optimal chair design for these athletes. As the organism level constraints for each athlete may change somewhat over time due to training, injury or the effect of neurodegenerative diseases, continuing assessment of chair design should be performed. The interested reader should also consult Burkett and Mellifont (2008) who used a similar theoretical approach when seeking to improve the performance of the six-strong Australian cycling team at the 2004 Paralympic Games by testing the effect of a number of potential changes in bike setup.

#### Training

Strength and conditioning training is an important component for most Olympic sports, with many more Paralympic sports also starting to utilise this mode of training in order to reduce the organism constraint of their disability. While a variety of exercise modes have been shown to significantly increase muscular strength and power and aerobic fitness in non-athletic individuals with a disability [27], very little of this research has been conducted with Paralympic wheelchair athletes or examined changes in functional performance [3,28]. The lack of studies in this area utilising athletic individuals is a limitation as Dallmeijer et al. [28] have demonstrated that the response to wheelchair rugby training is much lower in Paralympic athletes than non-athletic individuals with a disability. This means that the results of the studies using non-athletic individuals with a disability may not necessarily directly apply to Paralympic athletes and that more specific strength and conditioning programmes that factor in the interaction of the three levels of constraint may need to be developed.

Turbanski and Schmidtbleicher [3] observed improvements in selected kinetic and kinematic measures after an 8 week resistance training programme involving moderately heavy bench press exercise. They found that wheelchair athletes demonstrated significant improvements in strength and power parameters as a result of resistance training and that these effects were comparable to control subjects without spinal cord injury. Specifically, maximal velocity measured from a bench press throw improved from 2.39 to 2.49 m/s, and maximum bench press acceleration increased by 24.6%. Maximum force increased by 31.6% and maximum rate of force development demonstrated an impressive enhancement of 71.5%. These contributed to a trend for an improvement in 10 m sprinting performance (6.2%) that approached statistical significance (p = 0.058) [3]. These greater increases in strength and power in weight training movements than sprinting performance appears consistent with the review of Cronin et al. [29] for able-bodied sprinters, whereby increases in squat strength of ~23% were required for significant improvements in sprinting ability of > 2%. Although the lack of a comparable control group of athletes with a disability was a limitation of the Turbanski and Schmidtbleicher [3] study, these Paralympic athletes were very familiar with performing short sprints during training and competition. Therefore it would appear very unlikely that any learning effect occurred, meaning that the non-significant 6.2% improvement was likely to be a true change in performance and that the lack of statistical significance reflected a lack of statistical power of the small sample size.

#### Injury

Injury to the individual will act as an organism level constraint that may alter movement patterns, reduce subsequent performance and further increase the risk of future injuries to the same area. Many studies have described the importance of studying propulsion biomechanics to prevent injury not only in sport performance but also in daily life for wheelchair users. Since Paralympic athletes use their upper extremity for all mobility and daily functional tasks (with many of these individuals using wheelchairs for their mobility needs), an injury to the upper extremity from sport use can be detrimental to all activities of daily living and their overall quality of life [5,22,30].

The shoulder girdle is the primary source of power in most activities performed by wheelchair users but it is designed for freedom of movement rather than repetitive loading so manual wheelchair users are prone to many injuries of the upper limb, especially to the shoulder [4,6]. According to Ferrara and Peterson [8], the location of Paralympic injuries appears to be sport and disability dependant, with upper extremity injuries more frequent in athletes who utilise wheelchairs for their mobility needs. This was supported by Nyland, Snouse, Anderson, Kelly and Sterling [7] who found that in a group of USA Paralympians the wheelchair athletes had higher rates of shoulder, elbow-arm and forearm-wrist injuries compared to other Paralympian athletes. Unfortunately Nyland et al. [7] did not provide a full epidemiological description of these injuries, meaning that it is difficult to determine how the interaction of the intrinsic (organism) and extrinsic (task and environmental) constraints of wheelchair propulsion may have influenced these injury rates.

However, there is some evidence that the stroke patterns used by wheelchair athletes may be associated with injury risk [31,32]. For example, larger end of ranges of motion of the propulsion phase where peak shoulder forces and moments are shown to occur [4,6], frequent starting and stopping, and regularly propelling on outdoor and inclined surfaces [33] are risk factors for upper limb injuries because of increased and cumulative loading on the upper limb. Further biomechanical studies that determine how factors such as the position of the athlete in the wheelchair influence the overall shoulder stresses may help identify harmful aspects to a manual wheelchair user’s stroke patterns. This information may be used to design training programmes to reduce injury risk and/or be used for wheelchair athletes undergoing shoulder rehabilitation [6,22].

Although included in the organism level constraint section, we must reinforce the importance of how the three levels of constraints interact to alter the injury risk (by example, altering stroke patterns and mechanics) and that injury risk is not isolated to the organism alone. To gain more insight into the determinants of injury risk, an examination of the interaction of intrinsic (organism) and extrinsic (task and environmental) constraints need to be considered. Some of the task and environmental constraints that may potentially have an effect on an individuals’ injury risk and performance are in the sections to follow.

### Task constraints

Wheelchair athletes participating in sports such as basketball, rugby and tennis would encounter a range of task and environmental constraints that differ to those encountered by track athletes, as they must frequently accelerate and decelerate, change direction at speed and perform sport-specific skills to adjust to the task constraints during the course of a game. It would appear likely that these differences in constraints encountered would alter hand rim propulsion biomechanics in ways that may impact on the overall training required to maximise performance as well as the injury risk to the upper extremities. Differences and similarities in wheelchair propulsion at various speeds between wheelchair racing and court sports will therefore be covered in the following sections.

#### Wheeled sports

##### Racing

**Figure 5:**
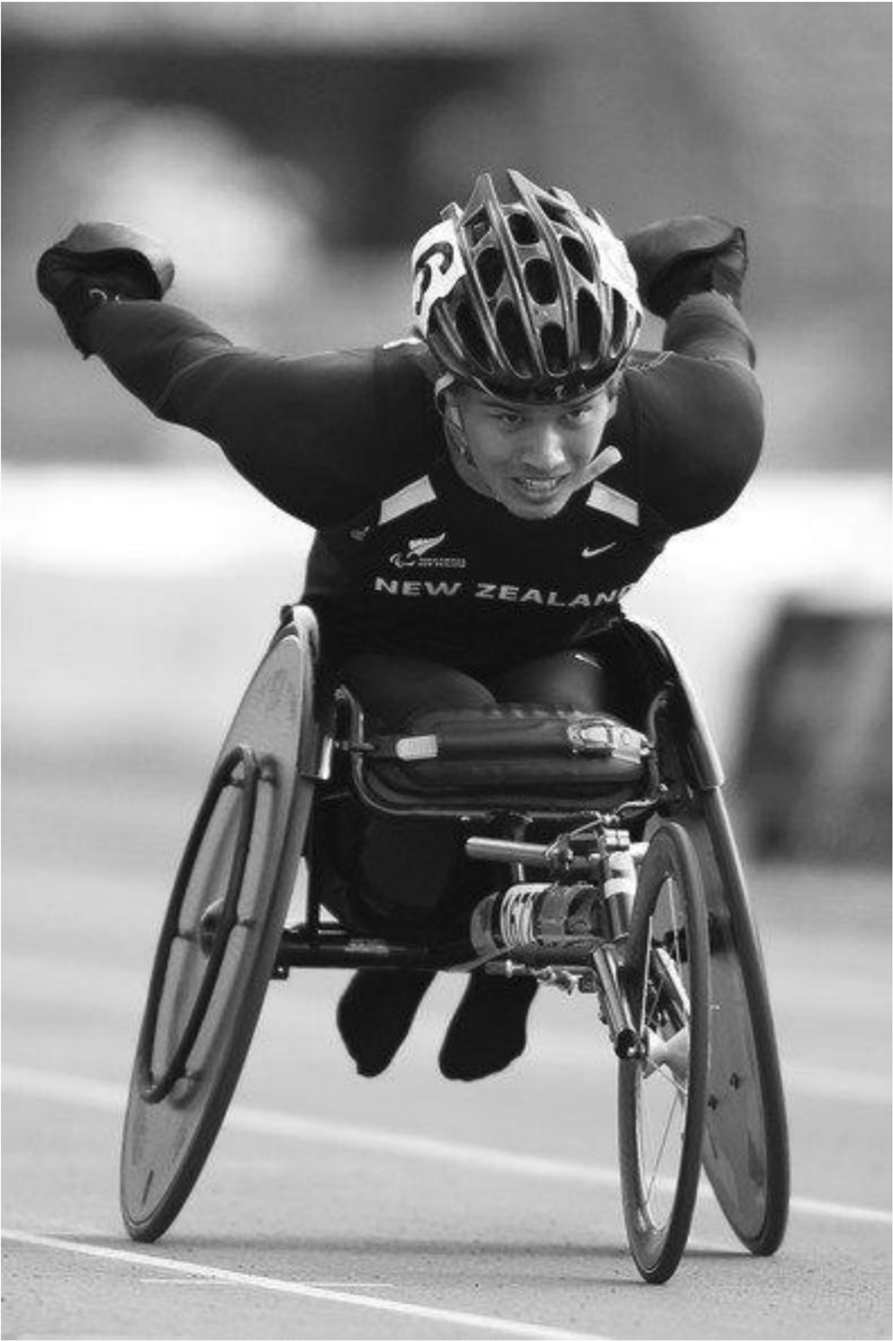
An example of a class T54 wheelchair racer.

The most important goal for wheelchair racing is to obtain and sustain a greater average velocity than your opponents. An example of a class T54 wheelchair racer is shown in Figure 5.

Unsurprisingly, a number of studies [4,6,34,35] have indicated that many kinematic and kinetic measures increase with speed. For example, Wang, Vrongistinos, & Xu [34] found that over consecutive pushes during wheelchair sprinting, higher range of motion values occurred around the shoulder and elbow leading to greater maximum angular velocity of the upper arm and forearm and an increased range of motion over which angular acceleration could be produced. In comparing the speed and stroke cycle characteristics during the 100 m wheelchair race, Chow [35] also found significant differences in stroke speed, length, push and recovery times at different portions of the race. At a faster speed, Boninger et al. [4] found that the elbow range of motion and peak acceleration in shoulder sagittal flexion and extension, abduction and adduction all increased. During these maximum accelerations the resultant force which reflected the limbs’ inertia, as well as the active contraction of the muscles, was found to increase at higher speeds.

In order to obtain maximum speed (momentum), the athlete must be able to apply an increasingly large propulsive force to the hand rim over an ever-decreasing period of time to achieve a high net impulse [9,36] and to adapt their stroking patterns to the variations in the constraints encountered as speed increases. In support of this view, O’Connor et al. [37] found that a longer hand contact time period and a slower wrist velocity would create a decelerating force and lead to a loss of wheelchair velocity at higher speeds. A higher wrist velocity might indicate that the hand is travelling as fast or faster than the hand rim, which would decrease application of a decelerating force to the push rim [37]. Due to this impulse – momentum relationship, Keogh [9] argued that high levels of strength and rate of force development would appear crucial for wheelchair sprinters. However, as stated previously very little strength and conditioning research has been conducted on Paralympic athletes, especially those involved in wheelchair sprinting, meaning much more research in this area is required.

##### Court sports

Unlike wheelchair racing where the specific movement dynamics may involve starting, accelerating, steady-state wheeling and deceleration phases, the task constraints in wheelchair ball sports like basketball, rugby and tennis also involve braking, change of direction, striking, catching, physical contact with opponents and/or blocking [32].

Goosey-Tolfrey et al. [38] found that tennis players had significantly reduced acceleration and maximum velocities during the first three pushes while holding a racquet in contrast to trials done without a racquet, as holding the racquet interfered with the hand contact on the rim. Such a result suggests there is potential for wheelchair tennis players to experiment with different ways to hold their tennis racquet while accelerating that allow more efficient transfer of muscular forces to the hand rim. Coutts [24] showed that compared to track athletes, basketball players had a significantly faster wheelchair velocity during the first push (achieving 80% of their peak speed during the third push) and similar speeds during the second and third pushes but that track athletes attained a higher peak speed during a 10 second effort.

The results of these studies examining the effect of different task constraints type of sport (racing vs court) and indirectly the effect of speed have a number of implications. They suggest that to develop sufficient levels of functional variability to perform well in different competitive conditions and tasks and minimise injury risk, Paralympic athletes need to train utilising some variations of the three levels of constraint that they will likely encounter in competition. If such specific training is not performed e.g. wheelchair rugby players only performing straight line rather than change of direction sprinting or training not reflecting the time-motion characteristics of the sport, performance will be reduced and injury risk likely increased.

### Environmental constraints

A variety of environmental constraints, for example increasing resistive forces, may pose a range of difficulties for those in hand rim propelled wheelchairs. Although wind, temperature and humidity may all be environmental constraints that influence wheelchair propulsion, the friction of the surface beneath the wheelchair and between the user and the wheelchair as well as the incline of the surface appear to be the most commonly assessed environmental constraints in this area [22]. The literature has confirmed that users often use functional variability by changing their propulsion biomechanics to suit the variations in these environmental conditions [30,33], with the likely effect of this being to overcome these difficulties and again, prevent injury.

#### Friction and surface inclination

Friction is an important type of force between the user’s hand and the hand rim as well as the wheel and ground that will have an impact on wheelchair sport performance and injury risk. For example, a sufficient amount of axial force, the portion of the total force that is directed toward the axle, is needed to help maintain friction between the hand and the hand rim, otherwise the hand may slip [22,37].

Goosey-Tolfrey (2010) found that because of the limited hand function that wheelchair rugby players often possess, these players may need to use adaptive equipment in the form of gloves and or hand rim modifications to increase the hand-hand rim friction so to improve their ergonomics of propulsion. Research by Coutts [24] supported this view, findings that basketball players with low levels of disability use their bare hands, while track athletes with less function wore gloves and used push rims that were coated with tape and/or adhesive compounds. The track athletes had greater positive and negative accelerations which were believed to be due to differences in hand to push rim surface friction.

Koontz et al. [33] conducted a kinetic analysis of manual wheelchair propulsion during start up on select indoor and outdoor surfaces that included high- and low-pile carpet, indoor tile, interlocking concrete pavers, smooth level concrete, grass, hardwood flooring, and a sidewalk with a 5^o^ ramp. In comparison to smooth level concrete, the kinetic requirements of the start-up phase increased substantially across the other surfaces. For example, the peak wheel torque and peak resultant force for the 5^o^ ramp (3.51 and 3.54 times), grass (3.11 and 2.44 times), and interlocking pavers (2.59 and 2.38 times) were significantly greater than the smooth concrete surface and many of the other low-friction, flat surfaces. This was likely due to the incline of the ramp and the greater friction imposed by the grass and interlocking pavers [33]. Using surfaces with greater friction or small inclines could therefore be used as a more specific resistance training tool than gym-based exercises for manual wheelchair users, as is done with sled towing for increasing able bodied sprinting speed [39,40]. This is reflected in the results of Richter et al. [30], who found that peak hand rim forces increased markedly with increasing grade, with an average 218% increase from the level to a 6° grade condition. These results would suggest that high ground friction or incline wheelchair training should be progressed slowly and involve relatively low volume as this form of training would lead to increased joint loading and may increase the likelihood of developing upper limb injuries.

**Figure 6:**
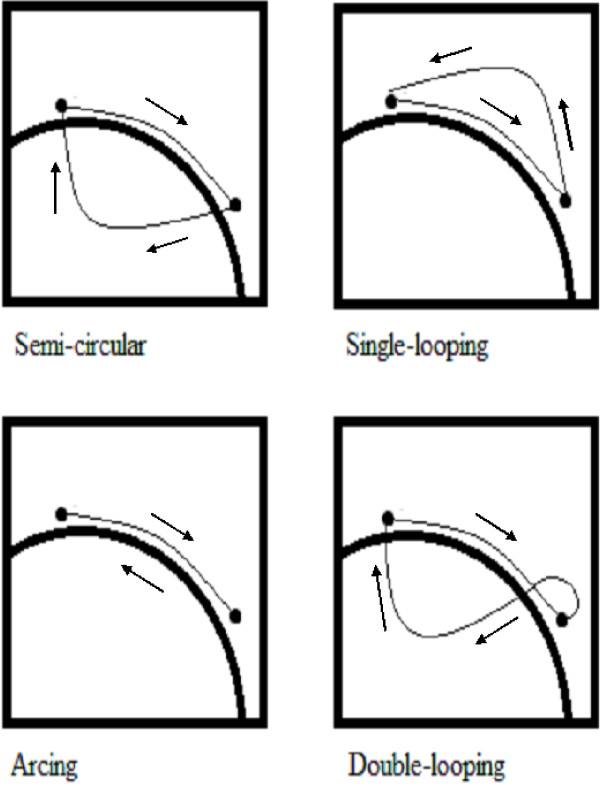
**Stylized illustration of stroke pattern classification during wheelchair propulsion.** The hand contacts the rim at the top, is constrained to follow the hand rim during the push but the user is free to choose how to follow through between pushes. In the arcing pattern, the user’s hand travels back along the hand rim between pushes (adapted from Richter et al. [24]).

The study by Richter et al. [30] also documented that by varying the inclination of the surface, some differences in the stroke characteristics emerge. These stroke characteristics have been classified as being semi-circular, single looping, double looping, and arcing (Figure 6) [30]. While a variety of stroke patterns were used for propulsion on a level surface, at 3° incline the stroke pattern with highest speed and peak force was arcing, whereas single looping had the greatest push angle and double looping had the highest push frequency. At 6° incline, the speed, push angle and push frequency all decreased and peak force increased for all patterns, with the arcing stroke pattern again having the highest values for all of these kinematic and kinetic variables [30]. Relatively little is known about how these variations in stroke patterns may occur naturally within and across different wheelchair events and in athletes with different degrees of disability. Thus, the alterations in surface friction and/or inclination seen in these studies may require the athletes to develop greater functional variability in their stroke patterns. The astute coach should therefore monitor the potential acute and chronic changes in wheelchair propulsion technique with variations in the environmental constraint so to ensure that such variability in technique is adaptive and functional rather than detrimental to performance and injury risk.

### Specificity using the constraints approach

The constraints-led approach suggests that to get the desired result and reduce injury risk during training, the organism, task and environment constraints of the movements performed in training should be similar to what is expected in competition. As biomechanics aims to provide insight into the underlying causes of human movement and to relate these causes to performance, and consequences such as injury, human movement should be studied under realistic constraints to action. On this basis, simulating manual wheelchair propulsion on lab-based ergometers can then be considered a deflection from reality which may alter the movement and/or performance and consequently the relationship between the parameters under study [32,36]. The two main specificity issues that are seen in research are the use of able bodied subject groups and using laboratory ergometers.

The laboratory ergometer has been used in many research studies examining wheelchair propulsion [5,6,31,37,41] as it has allowed detailed and repeatable physiological and biomechanical studies to be conducted on sedentary and expert wheelchair users, as well as evaluation of propulsion technique and mechanical strain [42]. However such an approach has its limitations. Moss et al. [36], Stankovits et al. [22] and Vanlandewijck et al. [32] all stated that this approach ignores the importance of environmental and task constraints such as the changes in air resistance brought about by the velocity and frontal surface area of the wheelchair-athlete interface, and internal and external forces acting on the wheelchair-user system, which will influence rolling friction and wheelchair balance. Since backward tilting is prevented on most stationary ergometers, the forces generated on the hand rim will also differ compared with the same task under field conditions, especially during the acceleration phase of a sprint task [32]. Inertial forces acting on the wheelchair, caused by accelerations and decelerations of the trunk and arms [32,36] are also reduced during ergometry assessments.

The predominance of lab-based ergometers studies may be due to historical constraints in instrumentation [43]. However, sophisticated biomechanical and physiological measurements outside the lab and on the track are becoming more feasible due to the further development and miniaturisation of monitoring devises, portable metabolic analysers and motion sensor technology [21,43]. Especially in wheelchair sports, this opens up new avenues for performance related research, although the consistency of the environmental conditions may need to be considered in order to ensure sufficient reliability of the acquired data [42].

A number of researchers [22,32] have stated that during wheelchair propulsion, a very specific activity is performed by a very specific group of users. So, as able bodied persons have different organism constraints that would likely not accurately represent the movement, force and muscle activity patterns used by experienced wheelchair users [32], they should not be used if possible in studies of wheelchair propulsion. Subsequently, studies such as Hintzy et al. [41] that used able bodied females to examine the force-velocity characteristics of maximal wheelchair sprinting and Lutgendorf et al. [44] who examined the use of different forms of gloves for wheelchair rugby movement may not be representative of results that would be obtained with Paralympic athletes.

## Conclusion

As the number of wheelchair users who are participating in wheelchair sport is growing worldwide, it is important to increase the understanding of the biomechanics of wheelchair propulsion to improve performance and decrease the risk of injury. The constraints-led approach is a useful tool when examining the outcome and coordination of a hand rim wheelchair user’s movement, and will assist the Paralympic athlete and coach better develop their overall training programme.

No single constraint can be considered in to isolation, so how the three levels of constraint interact is paramount to understanding wheelchair propulsion performance and injury risk during sport. The interaction of organism constraints such as players’ disability, classification level and chair set up will likely be important factors to consider when developing the appropriate training programme required by a wheelchair athlete to perform at their best whilst also reducing the risk of injury.

It is evident that while wheelchair athletes in racing and court sports all need to accelerate and achieve high speeds, they have very different task constraints that need to be considered, along with environmental constraints such as friction both between the user and wheelchair and wheelchair and ground. Unfortunately, very little empirical evidence is available on what constitutes appropriate training for these different groups of Paralympic athletes’ and how they may use functional variability when adapting their propulsion biomechanics to the variations in environmental conditions.

It is clear that much of the literature is almost 20 years old, based on using wheelchairs on ergometers, and even using able bodied individuals as subjects. Much research still needs to be done to fully understand the biomechanics involved in hand rim wheelchair propulsion in sport especially with the ever evolving wheelchair technologies. This research needs to use the appropriate subjects, be performed in ecologically valid, real-world environments and investigate the vast array of wheelchair sports that are now available.

## Competing interests

No authors have any competing interests.

## Authors’ contributions

ED and JK both made substantial contributions to: 1) conception and design of this review, including sourcing and reviewing relevant articles; 2) have been involved in drafting the manuscript or revising it critically for important intellectual content; and 3) have given final approval of the version to be published. Both authors read and approved the final manuscript.

## Authors’ information

ED has a Bachelor of Sport Studies from Massey University and is studying towards a Post Graduate Certificate in Sport and Exercise at AUT University, New Zealand. She is currently working at Sport Bay of Plenty in Tauranga.

JK has recently left AUT University in 2011 to take up a new position at Bond University, Australia. His primary teaching areas are biomechanics, motor control and motor learning. He was the Paralympics New Zealand Powerlifting team coach from 2007–2010.

## Pre-publication history

The pre-publication history for this paper can be accessed here:

http://www.biomedcentral.com/2052-1847/5/3/prepub
